# Ce(III)-MOFs with 1D chain architectures and their derivatives as earth-abundant electrocatalysts for the acidic oxygen evolution reaction

**DOI:** 10.1080/14686996.2026.2694962

**Published:** 2026-07-02

**Authors:** Gearóid Manning, Éadaoin Whelan, Róisín Maguire, Friedrich W. Steuber, Karl Ackland, Nianyong Zhu, Sebastien Vaesen, Lei Zhang, Wolfgang Schmitt

**Affiliations:** a School of Chemistry, Trinity College Dublin, University of Dublin, Dublin, Ireland; b Research Ireland Centre for Advanced Materials and Bioengineering (AMBER), Trinity College Dublin, University of Dublin, Dublin, Ireland; c Centre for Research on Adaptive Nanostructures and Nanodevices (CRANN), Trinity College Dublin, University of Dublin, Dublin, Ireland; d Institute of Modern Optics, College of Electronic Information and Optical Engineering, Nankai University, Tianjin, P. R. China

**Keywords:** Metal-organic frameworks, cerium, water oxidation, electrocatalysis

## Abstract

Cerium(III)-based metal-organic frameworks (Ce-MOFs) with one-dimensional chain secondary building units were synthesised and evaluated as oxygen evolution reaction (OER) electrocatalysts in acid. The MOFs act as pre-catalysts, transforming in-situ into active cerium oxide species. The best pristine MOF material that employs 1,3,5-triazine-2,4,6-triyl)tris(thiophene-2-carboxylate linkers (H_3_TTT), shows higher intrinsic activity and stability than commercial CeO_2_. The results highlight the role of linker chemistry. Pyrolysis at 800°C generates conductive CeO_2_-carbon composites with strong cerium anchoring and high sp^2^-carbon content. The thiophene-based MOF uniquely yields Ce_2_S_3_ and bulk defective CeO_2-x_ phases *via* in-situ sulfurisation. The derived materials exhibit lower overpotentials, smaller Tafel slopes, and improved durability, with Ce-TTT-derived material exhibiting a Tafel slope of 56 mV dec^−1^ and displaying stable activity after 1,000 CV cycles in 0.5 M H_2_SO_4_. These results identify Ce-MOFs as effective templates for an earth-abundant OER catalysts and highlight pyrolytic carbon matrices and phase evolution as key design parameters.

## IMPACT STATEMENT

Cerium-based MOFs act as tunable pre-catalysts that transform into active, durable OER materials under acidic pH conditions, while offering a scalable, earth-abundant pathway enabled by linker-controlled phase evolution and conductive carbon formation.

## GRAPHICAL ABSTRACT

**Figure:**
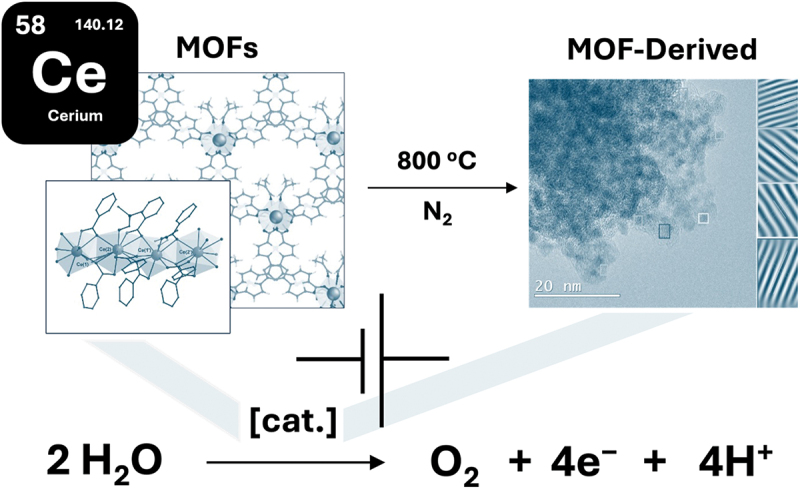

## 1. Introduction

Key to climate change mitigation and upholding the Paris Agreement commitment (stopping global temperatures increasing by more than 1.5°C, or at least less than 2°C compared to preindustrial levels), is the timely transition to a carbon neutral global economy [1,2]. The electrochemical splitting of water to generate hydrogen, using renewable energy sources, represents an ideal way of storing energy in chemical bonds of a non-carbon fuel with high gravimetric energy density [3]. So far, deployment of such technology has been limited by reliance on expensive platinum-group based materials as robust and efficient catalysts [4]. The water oxidation half-reaction, also known as the oxygen evolution reaction (OER), is a highly endergonic 4-electron reaction that requires large overpotentials (η) to achieve satisfactory oxygen evolution rates. As a result, OER is considered the limiting step in the overall water splitting reaction [5]. Significant progress has been made developing earth-abundant materials as OER electrocatalysts in alkaline media. However, alkaline water splitting technologies have a number of disadvantages such as relatively low current densities, hindered by OH^−^ transport through the anion-exchange membrane [5]. Greater current densities can be reached under acidic conditions due to fast proton transport through a proton exchange membrane (PEM) and high proton concentration for hydrogen generation (HER) [5,6]. However, only iridium or ruthenium-based materials are regarded to act as robust and efficient electrocatalysts under these conditions [5–7].

Various classes of metal-organic compounds, including oxo-clusters, metal-organic frameworks (MOFs) and metal-organic polyhedra (MOPs) represent promising compounds to mimic the efficacy of enzymatic systems (such as the oxygen-evolving complex of Photosystem II) due to their diverse and tuneable properties, allowing the exploration of structure–reactivity relationships to further improve catalytic performance [8–11]. MOFs are a class of porous coordination polymers, consisting of metal ions or clusters linked together by multitopic ligands into a two- or three-dimensional network with long-range ordering [12–16]. Due to their chemical and structural versatility, as well as their high surface areas, MOFs have found application as catalysts [17–20], materials for gas storage [21] and separation [22,23] and as sensors [24,25]. MOFs advantage over other porous materials such as zeolites, is that their properties can be tuned for an application by varying the linkers and/or the metal ions in the structure [26]. MOFs can however suffer from some drawbacks such as stability issues and low electronic conductivity. This may be mitigated via controlled pyrolysis of the MOF to achieve the synthesis composite of carbonous material and metal nanoparticles, while maintaining some of the MOF’s structure and porosity [27–30].

Cerium is the most abundant of the lanthanides, and the 25th most abundant element in the earth’s crust, with an abundance on par with copper and zinc, and nearly 6.7 × 10^4^ times more abundant than iridium or ruthenium [31]. Cerium dioxide (CeO_2_, ceria) is well known for its catalytic properties, with established uses such as in three-way catalytic converters, as well as being used as a catalyst/cocatalyst in energy conversion systems (both photocatalytic and electrocatalytic) [32,33]. Ceria’s catalytic properties derive from the reversible Ce^3+^/Ce^4+^ redox couple and consequent reversible surface oxygen anion exchange [33,34]. In recent years there has been growing interest in cerium-based metal-organic frameworks (Ce-MOFs) owing to the combination of tuneable microporosity, Ce^3+^/Ce^4+^ redox properties and the presence of low-energy 4f orbitals [35]. Potential application in fluorescence sensing, colorimetric sensing, catalysis and as templates for CeO_2_ nanostructures are described in the literature [32,35]. Ce(III)-MOFs based on tritopic ligands with trigonal geometry, commonly possess 1D Ce(III) SBUs linked in a way that creates 1D channels running parallel to the propagation direction of the SBU. This structure type is advantageous for gas adsorption, catalysis or as template for CeO_2_ nanostructures or composite materials [35]. To this end Cheng et al. explored the use of Ce-BTC MOF, [Ce(1,3,5-BTC)(H_2_O)_6_], as an electrocatalyst for the urea oxidation reaction (UOR). They found that controlled partial pyrolysis of Ce-BTC MOF in a nitrogen atmosphere resulted in ‘quasi-MOF’ material that maintained the porous structure of the precursor while increasing the number of active sites and greatly improving the catalytic activity toward UOR [36]. While CeO_2_ is well known for its catalytic properties and has often been employed as a co-catalyst for electrochemical water oxidation [37–42], there has been little exploration of cerium-based materials as primary electrocatalysts for the OER [43]. In particular, no examples of pristine Ce-based MOFs (or, more broadly, any lanthanide-based MOFs) have been reported as electrocatalysts for this reaction. This chapter seeks to explore this gap in the field.

**Figure 1.**
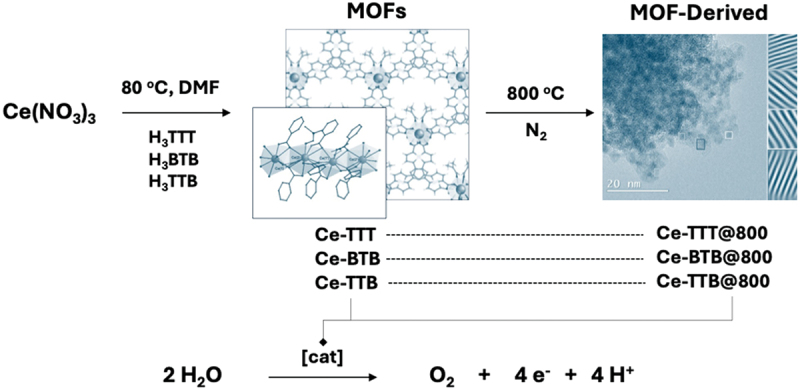
Synthetic approach to MOFs and pyrolysed, MOF-derived materials for the electrocatalytic oxidation of water.

**Figure 2.**
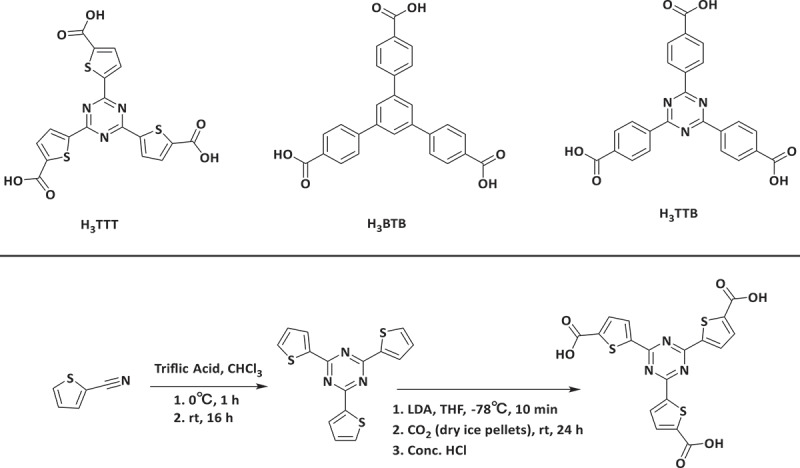
Top: chemical structures of tritopic organic ligands: H_3_TTT (5,5′,5″-(1,3,5-triazine-2,4,6-triyl)tris(thiophene-2-carboxylic acid)), H_3_BTB (4,4′,4″-(benzene-1,3,5-triyl)tris(benzoic acid)); H_3_TTB (4,4′,4″-(1,3,5-triazine-2,4,6-triyl)tris(benzoic acid)). Bottom: synthetic approach to H_3_TTT. LDA = lithium diisopropylamide.

Here we report the use of Ce(III)-based MOFs, constructed from tritopic ligands and featuring one-dimensional (1D) Ce(III) SBUs, as electrocatalytic or pre-electrocatalytic materials for the OER in acidic media. The effect of heteroatom substitution in the tritopic linker is explored through a comparative study of three Ce(III)-MOFs synthesised from structurally related ligands (Figures 1 & 2) with varying degrees of heteroatomic substitution. These ligands include 5,5′,5″-(1,3,5-triazine-2,4,6-triyl)tris(thiophene-2-carboxylic acid) (H_3_TTT); 4,4′,4″-(1,3,5-triazine-2,4,6-triyl)tris(benzoic acid) (H_3_TTB); and 4,4′,4″-(benzene-1,3,5-triyl)tris(benzoic acid) (H_3_BTB). The corresponding Ce-MOFs synthesised from these ligands are [Ce(TTT)(DMF)]_n_ (Ce-TTT), [Ce(TTB)(DMF)]_n_ (Ce-TTB), and [Ce(BTB)(DMF)]_n_ (Ce-BTB), respectively. Following, these Ce-MOFs were utilised as sacrificial templates for the synthesis of cerium-based electrocatalytic materials via controlled pyrolysis. It was anticipated that pyrolysis would generate CeO_2_ particles embedded in a conductive carbon matrix with the objective being to improve the OER kinetics on the catalysts while maintaining the activity and stability of their parent MOFs. This work highlights that Ce-MOFs can be used as effective pre-catalysts and structural templates for generating highly OER active, acid-stable CeO_2_-carbon composites. The results highlight the key roles of linker-derived carbon structure, nanocrystallite anchoring, and controlled phase evolution in governing catalytic activity and durability. Overpotentials at 10 mA cm^−2^ of 560 mV, 552 mV and 629 mV, and Tafel slopes of 56 mV dec^−1^, 83 mV dec^−1^ and 65 mV dec^−1^ were achieved for Ce-TTT@800/CP, Ce-TTB@800/CP and Ce-BTB@800/CP, respectively.

## 2. Experimental section

### 2.1. Materials

All chemicals, including HPLC-grade solvents, were purchased from commercial suppliers and were used without further purification. Millipore deionised water was used throughout the experiments.

### 2.2. Ligand synthesis

A novel tritopic linker, 5,5′,5″-(1,3,5-triazine-2,4,6-triyl)tris(thiophene-2-carboxylic acid), H_3_TTT, was synthesised in two steps (Figure 2), beginning with the acid-catalysed trimerisation of thiophene-2-carbonitrile to give 2,4,6-tri(thiophen-2-yl)-1,3,5-triazine, according to a previously reported synthesis [44]. Reaction of 2,4,6-tri(thiophen-2-yl)-1,3,5-triazine with lithium diisopropylamide (LDA) to give the trilithiated compound, followed by quenching with dry ice pellets and subsequent acidification, gave H_3_TTT with 60% yield. A detailed procedure for this synthesis is provided in Supplemental Information.

### 2.3. Synthesis of Ce(III)-MOFs

Ce-TTT: 5,5′,5″-(1,3,5-Triazine-2,4,6-triyl)tris(thiophene-2-carboxylic acid (H_3_TTT, 46.0 mg, 0.10 mmol) and cerium(III) chloride heptahydrate (37.25 mg, 0.10 mmol) were dissolved in DMF (8 mL) in a glass vial and sonicated for 10 min until homogeneous. The mixture was heated at 100°C for 48 h to afford yellow crystals, which were collected in *ca*. 77% yield of [Ce(TTT)(DMF)]_n_ (Ce-TTT) (52 mg). Ce-BTB: Following a previously reported procedure [45], 1,3,5-benzenetrisbenzoic acid (H_3_BTB, 0.438 g, 1.0 mmol) and cerium(III) chloride heptahydrate (0.373 g, 1.0 mmol) were dissolved in DMF (20 mL) in a Teflon-lined reaction vessel and sonicated for 10 min until a homogeneous mixture was obtained. A drop of triethylamine was added, and the mixture was further sonicated to homogenise the precipitate. The reaction was then heated at 120°C for 72 h, resulting in the formation of off-white crystals in *ca*. 85% yield of [Ce(BTB)(DMF)]_n_ (Ce-BTB) (0.651 g). Ce-TTB: 4,4′,4″-(1,3,5-Triazine-2,4,6-triyl)tris(benzoic acid) (H_3_TTB, 44.0 mg, 0.10 mmol) and cerium(III) chloride heptahydrate (37.25 mg, 0.10 mmol) were dissolved in DMF (8 mL) in a glass vial and sonicated for 10 min until a homogeneous mixture was obtained. The reaction mixture was heated at 100°C for 48 h, affording off-white crystals of [Ce(TTB)(DMF)]_n_ (Ce-TTB) in *ca*. 80% yield (52 mg).

### 2.4. Synthesis of pyrolysed MOF derivatives

**Figure 3.**
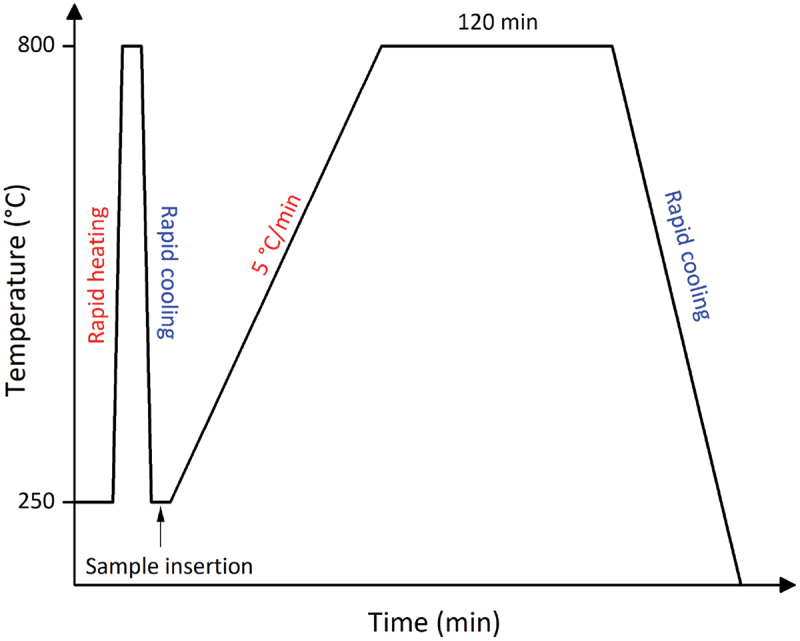
Heat treatment programme used for the pyrolysis of Ce(III)-MOFs under an inert nitrogen atmosphere. The tube furnace was first flushed by heating to 800°C and rapidly cooling to 250°C prior to sample insertion. Pyrolysis was carried out by heating Ce(III)-MOFs to 800°C at a rate of 5°C min^−1^, holding for 2 hours, followed by rapid cooling to room temperature.

Samples of the Ce(III)-MOFs, Ce-TTT, Ce-BTB and Ce-TTB, were pyrolysed in a tube furnace under an inert nitrogen gas atmosphere. The heat treatment programme employed is represented in Figure 3. Prior to sample insertion, the furnace was rapidly heated to 800°C and held at that temperature for 10 minutes, then quickly cooled to 250°C. This step was undertaken to remove any oxygen adsorbed within the tube. A crucible containing Ce(III)-MOF powder was then placed at the centre of the tube furnace, which was subsequently heated to 800°C at a rate of 5°C per minute. The furnace temperature was maintained at 800°C for 2 hours, after which the system was allowed to cool rapidly to room temperature. The resulting pyrolysed material, referred to as either Ce-TTT@800, Ce-BTB@800 or Ce-TTB@800 depending on the MOF precursor used, was recovered from the crucible.

### 2.5. Physicochemical characterisation

NMR spectra associated with the H_3_TTT preparation were recorded at 293 K on Bruker AVANCE III 400 MHz or AVANCE II 600 MHz spectrometers, with chemical shifts (δ, ppm) referenced to residual solvent signals. Single-crystal X-ray diffraction (SCXRD) data were collected on a Bruker APEX-II Duo diffractometer (Cu Kα, λ = 1.54178 Å) at 100 K, processed using the Bruker APEX suite, with absorption corrections applied via SADABS and structures solved and refined using SHELXT/SHELXL within *Olex2* [46–49]. FTIR spectra were acquired on a PerkinElmer Spectrum 100 ATR spectrometer (4000–350 cm^−1^, 1 cm^−1^ resolution), and Raman spectra were measured on a Renishaw 1000 micro-Raman instrument (785 nm excitation, calibrated to the Si 520 cm^−1^ band). UV-Vis absorption spectra were collected at room temperature on a PerkinElmer Lambda 35 spectrometer using 1 cm quartz cuvettes over 200–800 nm. Thermogravimetric analysis (TGA) was performed on a PerkinElmer Pyris-1 analyser under N_2_ (20 mL min^−1^) from 25–900°C at 3–5°C min^−1^. Powder X-ray diffraction (PXRD) patterns were obtained on a Bruker D8 ADVANCE diffractometer (Cu Kα radiation, 40 kV, 40 mA) over 4–80° (2θ). Electrospray ionisation mass spectra (ESI-MS) were recorded on a Micromass LCT time-of-flight instrument. SEM imaging was performed on a Zeiss ULTRA Plus microscope (10–20 kV) using samples drop-cast from IPA onto Si substrates and sputter-coated with Au-Pd; EDX spectra were collected with a 20 mm^2^ Oxford Inca detector. High-resolution TEM (HR-TEM) images were acquired on an FEI Titan 80 microscope operated at 300 kV, with lattice spacings determined via Fast Fourier Transform (FFT) analysis of individual crystallites and refined using inverse-FFT filtering to enable accurate line-profile measurements.

### 2.6. Electrocatalytic OER measurements

Heterogeneous electrocatalytic studies were carried out using an ALS-RRDE-3A rotating disk electrode (RDE) system coupled to a Biologic VSP potentiostat. A standard three-electrode configuration was employed, consisting of a Hg/HgCl (3 M KCl) reference electrode, a Pt wire counter electrode, and a modified carbon paste (CP) working electrode using 0.5 M H_2_SO_4_ aqueous electrolyte.

The CP electrodes were prepared by thoroughly grinding carbon paste with the desired amount of catalyst in an agate mortar. The resulting CP blend was packed into the electrode cavity (surface area: 0.07 cm^2^) and polished with satin weighing paper to ensure a smooth surface. For linear sweep voltammetry (LSV) and cyclic voltammetry (CV) experiments, the catalyst loading was 20 wt%. For chronopotentiometry measurements, a higher loading of 40 wt% was used, and the electrode surface was coated with 10 μL of Nafion 117 solution and allowed to dry prior to testing. For more information on OER measurements see Supplemental Information.

## 3. Results and discussion

H_3_TTT was synthesied through trimerisation of thiophene-2-carbonitrile under superacidic conditions to form a 2,4,6-tri(thiophen-2-yl)-1,3,5-triazine intermediate, followed by regioselective lithiation with lithium diisopropylamide (LDA) and subsequent carboxylation with carbon dioxide to yield the tris-carboxylic acid H_3_TTT. The preparation of Ce-TTT, was achieved under solvothermal conditions. This synthesis involved dissolving equimolar quantities of the novel ligand H_3_TTT and CeCl_3_.7 H_2_O in DMF and heating the solution at 100°C for 24 hours. This resulted in the formation of pale-yellow crystalline materials composed of aggregated, needle-shaped Ce-TTT crystals (Fig. S1). To study the effect of heteroatom substitution in the tritopic linker, the structurally related Ce(III)-MOFs, Ce-BTB and Ce-TTB were prepared under corresponding synthetic conditions using H_3_BTB and H_3_TTB, respectively (see Supplemental Information). It is well known that these synthetic conditions result in Ce(III) MOFs possessing 1D-chain inorganic SBUs [35].

### 3.1. Structural analysis

**Figure 4.**
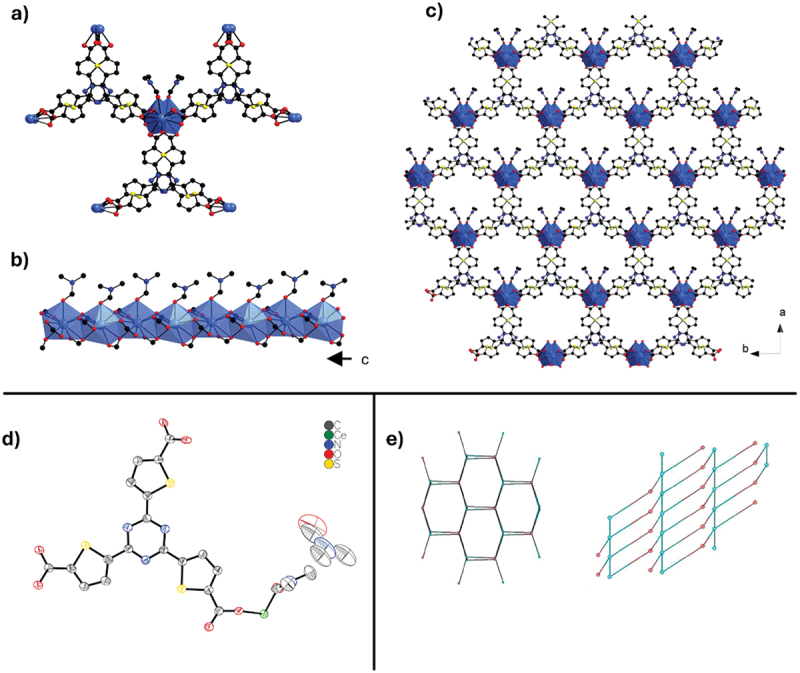
Representations of the crystal structure of Ce-TTT. (a) Structure of a 1D chain motif of Ce-TTT as viewed in the direction of the crystallographic c-axis; (b) Structure of the 1D chain motif in Ce-TTT, as viewed in the direction of the crystallographic a-b axis; (c) Extended framework structure of Ce-TTT with view in the direction of the crystallographic c-axis. DMF molecules squeezed from pores for clarity. d) ORTEP-style representation (50% probability ellipsoids) of the asymmetric unit of Ce-TTT; (e) Topological representation of Ce-TTT, as viewed along the crystallographic c-axis (left) and b-axis (right). Colour scheme: turquoise (Ce), red (TTT^3-^). Atom colour scheme for a)-c): black (C), red (O), blue (N), yellow (S), turquoise (Ce), H atoms omitted for clarity.

Separating a single needle crystal from aggregated clusters of crystals (Fig. S1) provided a sufficient size and quality crystal for single-crystal X-ray diffraction analysis. The structure of the Ce-TTT was identified as a porous three-dimensional framework structure, consisting of 1-D Ce(III) rod SBUs connected by TTT^3-^ carboxylate ligands (Figure 4). The crystal structure of Ce-TTT was solved and refined in the monoclinic space group *Cc*. The asymmetric unit of Ce-TTT contains one Ce(III) ion, one TTT^3-^ ligand and one coordinated DMF molecule (Figure 4c). The Ce(III) ions form an infinite rod shaped SBU, wherein the one dimensional chain of Ce(III) ions extends parallel to the crystallographic *c*-axis (Figure 4a). Each Ce(III) ion is ten-coordinated, with one bond to the oxygen atom of a coordinated DMF molecule and nine bonds to the carboxylate oxygen atoms of the TTT^3-^ ligands. Each carboxylate moiety of TTT^3-^ adopts a chelating bridging μ_2_-η^2^:η^1^ coordination mode whereby Ce(III) ions of 1D SBU are linked by the bridging carboxylates of the TTT^3-^ ligands. The Ce-Ce distance in Ce-TTT is 4.0896(3) Å. The chains of Ce(III) ions in Ce-TTT are close to linear, with Ce-Ce-Ce angles of 165.15(6) °, giving a slight zig-zag arrangement along the 1D SBU. An interesting feature of Ce-TTT is that the coordinated DMF molecules on successive Ce(III) ions bind from the same direction, giving rise to a chain of DMF molecules pointing into channels that extend along the *c*-axis. These topological aspects potentially facilitates accessible labile coordination site at each cerium atom rendering Ce-TTT interesting for electrocatalytic applications. The MOF forms a binodal 3,5-connected net with hms topology (Figure 4c). The stoichiometry of this net is (3-c)(5-c), and the net has point symbol {6^3^}{6^9^.8}. Each Ce(III) ion acts as a 5-connected node, as it is coordinated to three TTT^3-^ linkers and two other Ce(III) ions, and each TTT^3-^ linker acts as a 3-connected node. Crystallographic analyses indicate that the framework structure of Ce-MOF occupies ca. 65% of the unit cell volume; the largest channel in the structure is in the crystallographic c-direction, which can be penetrated by a sphere with 0.8 Å radius (if coordinated DMF molecules are removed). Powder X-ray diffraction (P-XRD) was used to confirm that the structure and phase purity of the as-synthesised Ce-TTT crystals were consistent with the reported crystal structure (see Supplemental Information).

**Figure 5.**
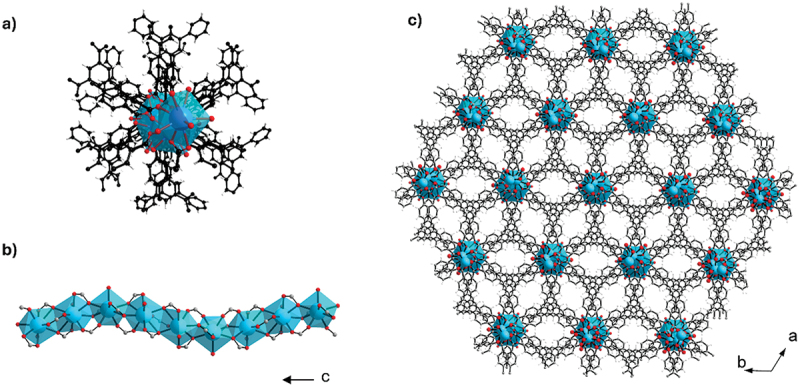
Representations of the crystal structure of Ce-BTB. (a) Structure of a 1D chain motif of Ce-BTB as viewed in the direction of the crystallographic c-axis; (b) Structure of the 1D chain motif in Ce-BTB, as viewed in the direction of the crystallographic a-b axis; (c) Extended framework structure of Ce-BTB with view in the direction of the crystallographic c-axis.

[Ce(BTB)(DMF)] MOF (Ce-BTB) crystals were synthesised following the procedure reported by Lin *et al*. [45] The identity of these crystals was confirmed by P-XRD analysis (see Figure S6 in Supplemental Information). Like Ce-TTT, the Ce(III) ions of Ce-BTB form an infinite rod-shaped secondary building unit (SBU), in which the one-dimensional chain of Ce(III) ions extends parallel to the crystallographic *c*-axis (Figure 5). Each Ce(III) ion is nine-coordinate, with one bond to the oxygen atom of a coordinated solvent molecule and eight bonds to the carboxylate oxygen atoms of the BTB^3-^ ligand. Each carboxylate group of the BTB^3-^ ligand adopts either syn-syn bridging or chelating bridging μ_2_-η^2^:η^1^ coordination modes [45].

[Ce(TTB)(DMF)] MOF (Ce-TTB) crystals were synthesised following the synthetic method employed for Ce-TTT (see Supplemental Information). The PXRD pattern of the resulting off-white crystalline powder (Ce-TTB) was found to closely match that of Ce-TTT (Fig. S7). The software package Expo2014 was used to fit the Ce-TTB experimental pattern to the Ce-TTT pattern, derived from single-crystal data, using the Le Bail method (Fig. S8) [50,51].

### 3.2. OER activity of pristine Ce(III)-MOFs

To investigate the potential of the Ce(III)-based MOFs as electrocatalysts for the oxygen evolution reaction (OER), heterogeneous electrochemical studies were conducted in 0.5 M aqueous H_2_SO_4_. This electrolyte was chosen as it is commonly used to simulate the acidic environment present in proton exchange membrane (PEM) electrolysers [52]. As Ce-TTT, Ce-BTB, and Ce-TTB all consist of one-dimensional (1D) Ce(III) chain secondary building units (SBUs), this structural similarity enables investigation of the effect of heteroatom substitution in the tritopic linkers. A commercially available CeO_2_ nano-powder (<50 nm) was used as a benchmark catalyst for comparison with the Ce(III)-MOFs in the electrocatalytic OER.

**Figure 6.**
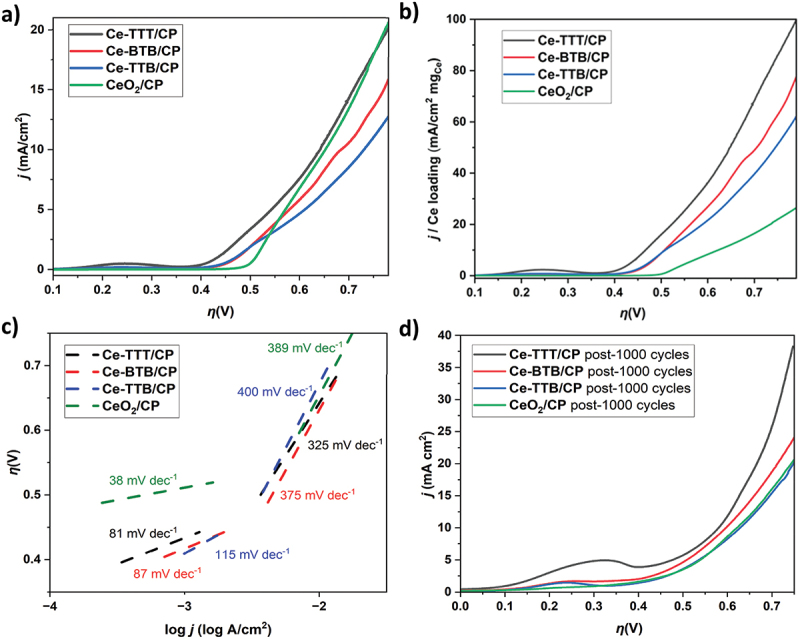
A) RDE-LSV plot of Ce-MOFs and CeO_2_ embedded in carbon paste (CP) electrodes. Measurements were performed in 0.5 M H_2_SO_4_ at a sweep rate of 1 mV s^−1^ with an electrode rotation speed of 1600 rpm. b) RDE-LSV plot of Ce-MOFs and CeO_2_ embedded in CP, normalised to loading of Ce. c) Tafel plot obtained from LSV data. d) RDE-LSV plot of Ce-MOFs and CeO_2_ embedded in CP, after 1,000 cyclic voltammetry cycles.

The kinetics of the electrocatalytic reaction and the stability of the cerium electrocatalysts under turnover conditions were evaluated using modified carbon paste (CP) electrodes as part of rotating disk electrodes in linear sweep voltammetry (RDE-LSV) experiments. Electrocatalyst-modified CP electrodes were employed instead of Nafion-based inks due to their superior electronic conductivity, which results in higher current densities. A catalyst loading of 20 wt% was chosen for optimal performance and mechanical stability. Figure 6a presents the RDE-LSV plots for each catalyst. For the three Ce(III) MOFs, a characteristic Ce(III) to Ce(IV) oxidation peak is observed at an overpotential (η) of approximately 250 mV (1.48 V vs SHE), in agreement with the literature [53]. This oxidation occurs prior to the onset of water oxidation, which begins above an overpotential of approximately 387 mV. This suggests that Ce(IV) represents the OER-active site and that the Ce(III)-MOFs function as pre-catalysts. The LSV for CeO_2_/CP lacks the Ce(III) → Ce(IV) oxidation peak, consistent with its initial Ce(IV) oxidation state.

A comparison of applied overpotentials (η) at various current densities is presented in Table 1. The onset overpotential (η), defined as the potential at which OER initiates, was estimated using the Tangent Method. Among the tested catalysts, Ce-TTT/CP exhibits the lowest onset η (387 mV), followed by Ce-TTB/CP (404 mV), Ce-BTB/CP (410 mV), and CeO_2_/CP (474 mV). A similar trend is observed at a current density of 1 mA cm^−2^. At 5 mA cm^−2^, the overpotential of CeO_2_/CP (609 mV) begins to intersect those of Ce-TTB and Ce-BTB. Notably, Ce-TTT/CP maintains superior performance across all current densities, being matched only by CeO_2_/CP at 20 mA cm^−2^. Given that all three MOFs pre-catalysts are constructed from one-dimensional Ce(III) chain SBUs, these results suggest that the variations in the properties of the tritopic linker can influence the onset characteristics and initial catalytic activity with respect to OER.

**Table 1.** Comparison of applied overpotentials (η) at various current densities for Ce-MOFs and pyrolysed Ce-MOFs derivatives, as well as commercial CeO_2_ nanoparticles benchmark electrocatalyst, embedded in carbon paste electrodes.

Electrode	η @ 1 mA cm^−2^	η @ 10 mA cm^−2^	η @ 20 mA cm^−2^	Tafel Slope (mV dec^−1^)	Stability (# cycles over potential window, h @ j)
Ce-TTT/CP	433 mV	644 mV	778 mV	81	1000 cycles 0.0–2.0 V *24 h@1 mA cm^−2^ *(40% wt. cat)
Ce-TTT@800/CP	487 mV	560 mV	633 mV	56	1000 cycles 0.0–2.0 V *24 h@1 mA cm^−2^ *(40% wt. cat)
Ce-TTB/CP	470 mV	731 mV	N.A.	115	1000 cycles 0.0–2.0 V *24 h@1 mA cm^−2^ *(40% wt. cat)
Ce-TTB@800/CP	442 mV	552 mV	621 mV	83	*24 h@1 mA cm^−2^ *(40% wt. cat)
Ce-BTB/CP	474 mV	685 mV	N.A.	87	1000 cycles 0.0–2.0 V *24 h@1 mA cm^−2^ *(40% wt. cat)
Ce-BTB@800/CP	506 mV	629 mV	756 mV	65	*24 h@1 mA cm^−2^ *(40% wt. cat)
CeO_2_/CP	510 mV	653 mV	774 mV	38	1000 cycles 0.0–2.0 V *24 h@1 mA cm^−2^ *(40% wt. cat)

When LSVs are normalised to Ce loading (Figure 6b) the activity difference between the Ce-MOF pre-catalysts and the CeO_2_ nano-powder become more apparent, with the former outperforming the latter at all OER overpotentials measured.

The kinetics of the oxygen evolution reaction (OER) on the various electrodes were studied by Tafel plot analysis using data derived from rotating disk electrode linear sweep voltammetry (RDE-LSV) (Figure 6c). The Tafel slope indicates the increase in overpotential required to achieve a tenfold increase in current density and is known to depend on the rate-determining step of the electrochemical process. Each electrode presented in Figure 6c exhibits two distinct Tafel regions, a lower overpotential region corresponding approximately to current densities of 1 mA cm^−2^ and a higher overpotential region corresponding approximately to current densities of *ca*. 10 mA cm^−2^. As the Tafel slopes of the lower overpotential region are below ~120 mV dec^−1^, this is considered to be the kinetic region of the curve and thus is reflective of the rate-determining step (RDS) within the OER mechanism [54]. The large apparent Tafel slopes measured in the higher overpotential region represent a mixed/intermediate regime, i.e. a convolution of kinetic and non-kinetic contributions, namely internal H_2_O gradients and confined O_2_ bubble effects within the microporous MOF/composite architecture, as well as potentially residual iR contributions [54–59]. Any mechanistic interpretations are restricted to the low-overpotential Tafel region, while the high-overpotential slope is reported as an apparent value reflecting the onset of these transport-related limitations.

In the lower overpotential region, Ce-TTT/CP and Ce-BTB/CP exhibit Tafel slopes of 81 mV dec^−1^ 87 mV dec^−1^, respectively, which are consistent with a chemical RDS (i.e. O-O coupling or O vacancy refilling) under non-ideal (Temkin) coverage of intermediates or competition between a chemical and an electron transfer limiting step [54–59]. Ce-TTB/CP exhibits a higher Tafel slope of 115 mV dec^−1^, which could be assigned to a rate-determining first electron transfer (i.e. initial water absorption/one-electron oxidation at active site) [54–59]. CeO_2_/CP displays more favourable kinetics than the Ce-MOFs, with a Tafel slope of 38 mV dec^−1^, consistent a chemical RDS [54–59].

**Figure 7.**
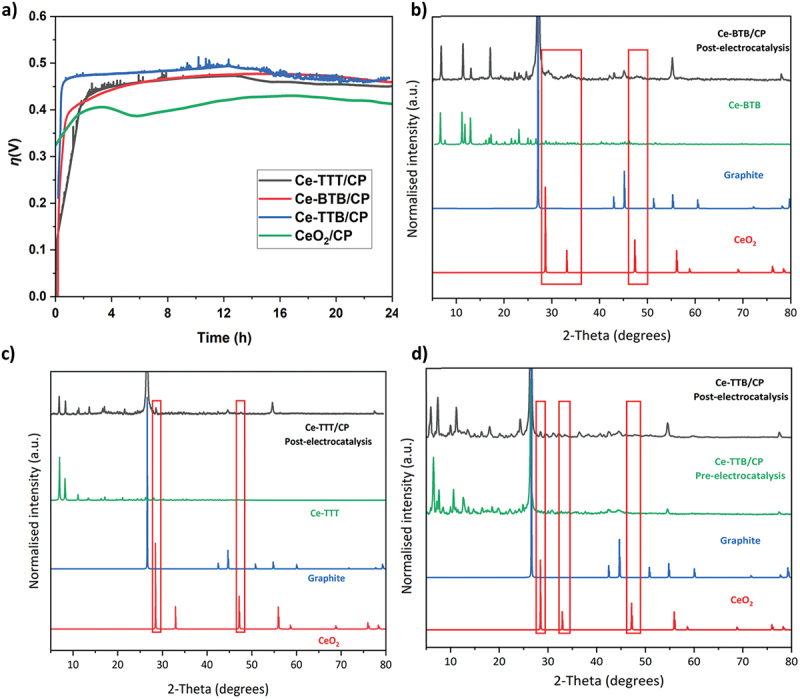
(a) Chronopotentiometry data for Ce-MOFs and CeO_2_ embedded in carbon paste (CP) working electrodes. Measurements were performed at a constant current density of 1 mA cm^−2^ in 0.5 M H_2_SO_4_ electrolyte at an electrode rotation speed of 1600 rpm. (b) PXRD pattern of Ce-TTT/CP after electrocatalysis (black), compared with calculated patterns for Ce-TTT (green), graphite (blue), and CeO_2_ (red). Red boxes indicate reflections shared with CeO_2_. (c) PXRD pattern of Ce-BTB/CP after electrocatalysis (black), compared with calculated patterns for Ce-BTB (green), graphite (blue), and CeO_2_ (red). Shared CeO_2_ reflections are highlighted in red. (d) PXRD pattern of Ce-TTB/CP after electrocatalysis (black), compared with calculated patterns for Ce-TTB (green), graphite (blue), and CeO_2_ (red), with red boxes marking reflections consistent with CeO_2_.

The long-term operational stability of the Ce-MOF-based electrocatalysts was further evaluated using 24-hour chronopotentiometry experiments (Figure 7a). A constant current density of 1 mA cm-^2^ was selected based on the Tafel analysis presented above. According to the Tafel plots, at this current density, the Ce-MOF-based electrocatalysts operate under kinetic controlled conditions. Consequently, any observed increase in the applied potential required to maintain 1 mA cm^−2^ can be attributed to electrocatalyst degradation rather than mass transport or other non-kinetic limitations.

All three Ce-MOF-based electrocatalysts appear to be stable over the 24-hour test period. For Ce-TTT/CP, the average overpotential between 3 and 24 hours is approximately 460 mV, reaching a maximum of 472 mV at 13 hours, after which the overpotential gradually decreases, reaching 450 mV at 24 hours. This slight decline in overpotential is consistent with the findings of prolonged CV cycling experiments in which OER activity improved with cycle number (discussed below). Extended electrochemical operation induces progressive transformation of the Ce(III)-MOF pre-catalyst into a Ce(IV)-based active species, resulting in a growing number of catalytically active sites and, consequently, improved performance. Similar behaviour is observed for Ce-BTB/CP and Ce-TTB/CP. With Ce-BTB/CP also having an average overpotential of approximately 460 mV, peaking at 477 mV at 13.7 hours before declining to 458 mV at 24 hours. Ce-TTB/CP is observed to have a slightly higher average overpotential of 490 mV, reaching a maximum value at 493 mV at 12.1 hours, before gradually declining to 467 mV at the 24-hour mark.

The commercial CeO_2_ nanoparticles, (CeO_2_/CP), included here as a benchmark, were also found to be relatively stable over the 24-hour test period, with the overpotential increasing only slightly from 407 mV at 3 hours to 412 mV at 24 hours.

Overall, the three Ce-MOF-based electrocatalysts displayed comparable behaviour, though the benchmark showed a moderately lower overpotential than the MOFs. This trend is consistent with their Tafel slopes at 1 mA cm^−2^: CeO_2_/CP exhibited the lowest slope (38 mV dec^−1^), followed by Ce-TTT/CP (81 mV dec^−1^) and Ce-BTB/CP (87 mV dec^−1^), and then Ce-TTB/CP with its slightly higher overpotential of correlating with its higher Tafel slope (115 mV dec^−1^). The fact that the Ce-MOF catalysts converge closely with the CeO_2_ nanoparticles further reinforces the idea that the Ce(III)-MOF act as a pre-catalyst for a cerium(IV)-oxide active species.

Powder X-ray diffraction (PXRD) analysis was carried out on Ce-MOF/CP electrodes used in the 24-hour chronopotentiometry experiments at 1 mA cm^−2^. In each case, the MOF-CP composite material was removed from the electrode holder after electrocatalysis and placed on a zero-background PXRD sample holder. The quality of the PXRD patterns were limited by the small amount of sample (<40 mg of MOF-CP composite) available.

The PXRD patterns of the Ce-MOF/CP electrodes after electrocatalysis (Figure 7) show that the parent MOF structures remain largely intact in the bulk, while new reflections are attributable to the formation of CeO_2_ at the electrochemically active interface. For Ce-TTT/CP (Figure 7b), the pattern is dominated by the Ce-TTT framework and graphite from the carbon paste, with additional peaks at ~28.5° and ~47.5° (2θ) indicating the formation of CeO_2_. Ce-BTB/CP (Figure 7c) similarly retains the characteristic Ce-BTB reflections, accompanied by weak, broad CeO_2_ peaks at ~29°, 33°, and 48°. An analogous behaviour is observed for Ce-TTB/CP (Figure 7d), where the post-electrocatalysis pattern matches that of the pristine material except for the emergence of CeO_2_ reflections at ~29°, 33°, and 48°.

These PXRD results indicate that the Ce-MOFs remain structurally intact in the bulk of the composite material following electrocatalysis. However, the emergence of CeO_2_-derived signals suggests that Ce-MOF particles located at the electrochemical interface undergo oxidation to form CeO_2_, which is likely to act as the catalytically active species during the oxygen evolution reaction.

To further assess the stability of the OER activity demonstrated by the Ce-based MOFs, each electrocatalyst was subjected to 1,000 CV cycles at a scan rate of 100 mV s^−1^, over a potential range of 0.2 V to 2.0 V versus SHE, in 0.5 M H_2_SO_4_ (Figs. S18a-S20a). A linear sweep voltammogram (LSV) was recorded both before and after the CV experiment, at a scan rate of 1 mV s^−1^ (Figures. 6d, S18b-S20b). All three Ce-MOF/CP electrodes exhibit enhanced OER activity after 1000 CV cycles in 0.5 M H_2_SO_4_ electrolyte. The magnitude of improvement is comparable across the series: at an applied overpotential of 700 mV, Ce-TTT/CP and Ce-BTB/CP show increases in current density by 54% and 53%, respectively, while Ce-TTB/CP demonstrates a 60% increase. In contrast, CeO_2_/CP displays no such enhancement in activity following CV cycling but maintains its initial performance. These results support the hypothesis that Ce(III)-MOFs function as pre-catalysts, undergoing partial transformation during cycling to generate OER active cerium oxide phase. The fact that catalytic activity improves for the Ce-MOFs (but not for the CeO_2_ nanoparticles) suggests that additional active sites become accessible as the MOF crystals progressively transform during repeated cycling.

### 3.3. Pyrolysed Ce(III)-MOFs

The electrocatalytic analyses demonstrated that Ce-TTT, Ce-BTB and Ce-TTB can function as effective pre-catalysts for the oxygen evolution reaction (OER) in acidic media, with the in-situ formation of catalytically active cerium (IV) oxide phases. Building on these findings, we explored their use as sacrificial templates for the synthesis of electrocatalytic cerium-based materials via controlled pyrolysis. It was anticipated that pyrolysis under N_2_ would generate CeO_2_ particles embedded in a conductive carbon matrix with the objective being to improve the OER kinetics while maintaining the activity of the parent MOFs. The pyrolysed samples were obtained through a defined, heat treatment programme as shown in Figure 3.

**Figure 8.**
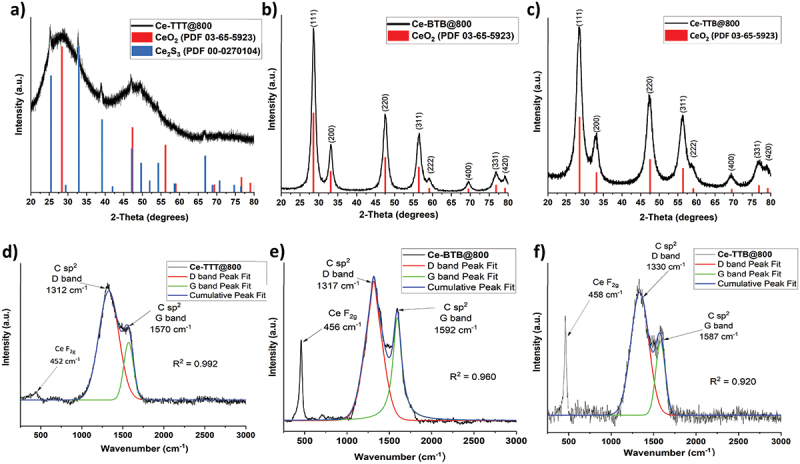
(a) PXRD pattern of Ce-TTT@800 (black), overlayed with ICDD reference standards for cerium sulfide (PDF 00–027-0104) in blue, and cerium oxide (PDF 03–65-5923) in red. (b) PXRD pattern of Ce-BTB@800 (black) overlayed with the ICDD reference standard for CeO_2_ (PDF 03–65-5923) in red. (c) PXRD pattern of Ce-TTB@800 (black) overlayed with the ICDD reference standard for CeO_2_ (PDF 03–65-5923) in red. (d) Raman spectrum of Ce-TTT@800 with deconvolution of the overlapping carbon D and G bands using a Gaussian peak fitting model. (e) Raman spectrum of Ce-BTB@800 with deconvolution of the overlapping carbon D and G bands using pseudo-Voigt peak fitting model. (f) Raman spectrum of Ce-TTB@800 with deconvolution of the overlapping carbon D and G bands using Gaussian peak fitting model.

Powder X-ray diffraction (PXRD) analysis of Ce-TTT@800 reveals that a composite material was formed by the pyrolysis of Ce-TTT. The PXRD pattern of Ce-TTT@800, presented in Figure 8a, features two broad reflections centred at 28.6° and 47.4°, which are characteristic of cerium oxide (CeO_2_) (111) and (220) planes. Superimposed on these broad peaks are smaller peaks at 25.2°, 32.8°, 39.0°, 47.1°, 49.5°, 54.1°, and 66.7°. Using the DIFFRAC.EVA software package, a database match was made between this pattern of peaks and International Centre for Diffraction Data (ICDD) reference standards for Ce_2_S_3_ (PDF 00–027-0104) and CeO_2_ (PDF 03–65-5923).

The findings of the PXRD analysis suggest that the pyrolytic decomposition of Ce-TTT results in the formation of both CeO_2_ and Ce_2_S_3_ phases. The formation of CeO_2_ under an inert (oxygen-free) atmosphere can be attributed to the MOF itself acting as an internal oxygen source, as each Ce(III) centre in Ce-TTT is coordinated to nine carboxylate oxygen atoms. It is proposed that, upon thermal decomposition of the inorganic SBU, the Ce(III) centres are oxidised to Ce(IV), leading to the formation of CeO_2_ nano-crystallites.

The formation of Ce_2_S_3_ is similarly attributed to the presence of sulfur within Ce-TTT, originating from its thiophene moieties. Under pyrolytic conditions, a sulfurisation reaction appears to occur, in which the decomposed thiophene units of the organic linker act as the sulfur source. To the author’s knowledge, this represents the first report of a MOF being used as a precursor for the synthesis of a lanthanide(III) sulfide phase. Hirai et al. previously reported the synthesis of Ce_2_S_3_ via sulfurisation of CeO_2_ powder using carbon disulfide (CS_2_) as the sulfur source and argon as the carrier gas at temperatures between 700 and 750°C [60]. Since CS_2_ is a known pyrolysis product of thiophene [61], it is therefore proposed that Ce-TTT undergoes a similar sulfurisation pathway upon thermal decomposition of the thiophene moieties, generating CS_2_ *in-situ*.

Furthermore, Hirai et al. found that the addition of carbon black to the sulfurisation reaction accelerated the formation of Ce_2_S_3_ *via* carbothermic reduction of CeO_2_. Given that the pyrolytic decomposition of Ce-TTT also yields carbon (as confirmed by Raman spectroscopy), a similar carbothermic reduction mechanism may also contribute to the formation of Ce_2_S_3_ in this case.

The PXRD patterns of Ce-BTB@800 and Ce-TTB@800 (Figures. 8b,c) both exhibit reflections characteristic of CeO_2_, matching the ICDD reference standard PDF 03–65-5923. Ce-BTB@800 shows peaks at 28.6°, 33.2°, 47.6°, 56.4°, 59.2°, 69.5°, 76.8°, and 79.2°, while Ce-TTB@800 displays analogous reflections at 28.5°, 33.9°, 47.4°, 56.3°, 59.1°, 69.4°, 76.7°, and 79.1°. In both cases, the peaks index to the (111), (200), (220), (311), (222), (400), (331), and (420) planes of CeO_2_, confirming the formation of fluorite-phase ceria in the pyrolysed materials.

The Raman spectra of the pyrolysed Ce-MOFs (Figure 8d-f) exhibit the characteristic carbon D and G bands, with Ce-TTT@800 showing features at 1312 and 1570 cm^−1^ and an I(D)/I(G) ratio of 1.89 (Gaussian fit), corresponding to ~ 94% sp^2^ and 6% sp^3^ carbon according to the Ferrari–Robertson relation [62,63]. Ce-BTB@800 and Ce-TTB@800 display similar D/G positions (1317/1592 and 1330/1587 cm^−1^, respectively), with I(D)/I(G) values of 1.35 and 1.68, corresponding to 68% sp^2^/32% sp^3^ and 84% sp^2^/16% sp^3^ carbon [63].

Combined Raman and PXRD characterisation indicates the *in-situ* sulfurisation process arising from pyrolysis of the sulfur-containing TTT linker in Ce-TTT@800, generates a bulk population of lattice defects (oxygen vacancies/ Ce^3 +^ centres) within the ceria phase. This contrasts with the surface-localised defect populations that characterise Ce-BTB@800 and Ce-TTB@800. This is evident from the Raman spectra (Figs. S30-S32). The Ce-O F_2_g mode red-shifts from ~465 cm^−1^ (for pristine CeO_2_ [64]) to 456 and 458 cm^−1^ for Ce-BTB@800 and Ce-TTB@800, respectively, while in Ce-TTT@800 this was red shifted to 452 cm^−1^.

Furthermore, the ceria defect-induced (D) band near ~600 cm^−1^, associated with oxygen vacancies in the fluorite lattice [65,66], is significantly more pronounced in the case Ce-TTT@800, with an I_D_/I_F2g_ ratio of 0.52 versus 0.006 and 0.018 for Ce-BTB@800 and Ce-TTB@800, respectively. The I_D_/I_F2g_ ratio was calculated using the integrated area of the F_2_g and D bands (more details in Supplementary Information). For comparison, the commercial CeO_2_ nanoparticles used in this study were found to have an I_D_/I_F2g_ ratio of 0.007. These observations are reflective of the lattice expansion accompanying Ce^4+^ to Ce^3+^ reduction (as the 1.14 Å eight-coordinate radius of Ce^3+^ is substantially larger than the 0.97 Å eight-coordinate radius of Ce^4+^) [67], a correlation well established for reduced ceria [68].

The moderate F_2_g red-shift and limited D band intensity of Ce-BTB@800 and Ce-TTB@800 are attributed to the combined effect of phonon confinement in ceria nanocrystallites [69] and surface population of defects stabilised by interfacial coupling with the sp^2^-rich carbon phase [70–74]. This surface localised defect assignment is consistent with the PXRD patterns of Ce-BTB@800 and Ce-TTB@800, which are very similar to each other and indistinguishable from pristine ceria, indicating the bulk fluorite lattice is undistorted.

**Figure 9.**
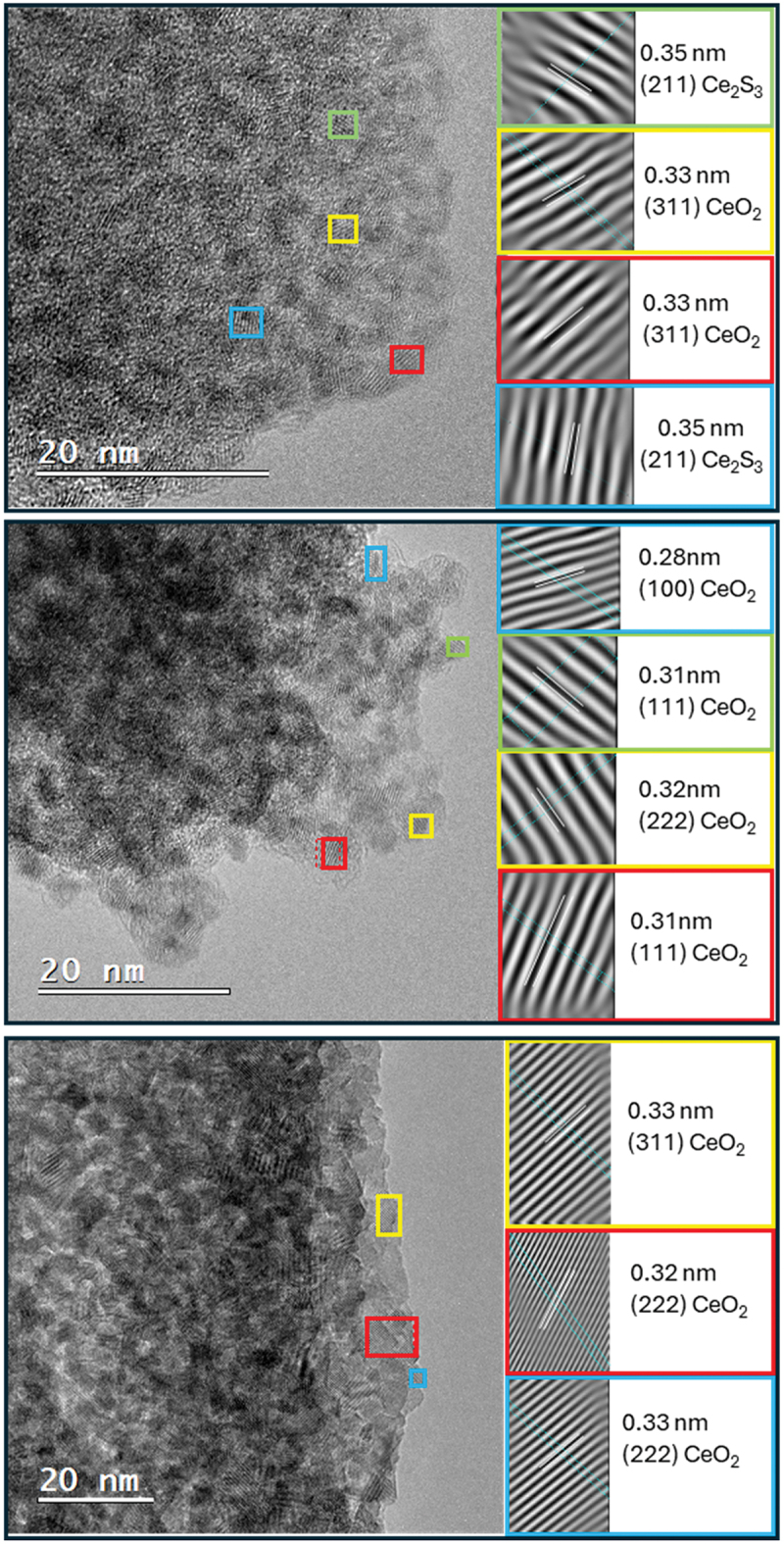
(a) HR-TEM images acquired at the edge of a Ce-TTT@800 particle, dominated by Ce_2_S_3_ and CeO_2_ crystallites. (b) HR-TEM images acquired at the edge of a Ce-BTB@800 particle, dominated by CeO_2_ crystallites. (c) HR-TEM images acquired at the edge of a Ce-TTB@800 particle, dominated by CeO_2_ crystallites.

In contrast, Ce-TTT@800 has an anomalously broadened F_2_g band (relative to its red-shift) and large I_D_/I_F2g_ ratio, which are indicative of a heterogeneous distribution of Ce-X environments [75–77]. This Raman signature corresponds with the substantially broader CeO_2_ PXRD reflections noted previously for this sample. As HR-TEM (Figure 9a) confirmed that crystallite sizes of ceria and cerium sulfide phases are comparable, size cannot be the primary source of the broadening of ceria PXRD reflections and therefore microstrain is identified as the dominant source of broadening [78]. These effects are consistent with the lattice distortion expected from the partial substitution of O^2-^ (1.38 Å) with the larger S^2-^ (1.84 Å) in the anion sublattice [67], together with the chemical expansion accompanying bulk Ce^3+^ formation [79].

High-resolution transmission electron microscopy (HR-TEM) was employed to probe the local structure of the pyrolysed Ce-MOFs. Ce-TTT@800 particles remained intact after sonication, retaining their high aspect-ratio morphology (Fig. S21). HR-TEM images of these edge regions (Figures. 9a and S22) reveal 2–3 nm crystallites. Fast Fourier transform (FFT) analysis (Fig. S27) indicates multiple surface phases. Figure S22 shows CeO_2_ crystallites interspersed with amorphous domains, identified by lattice spacings of 0.31 nm and 0.38 nm corresponding to the (111) and (110) planes, respectively. A distinct region on the same particle (Figure 9a) contains Ce_2_S_3_ (0.35 nm, (211) plane) together with CeO_2_ (0.33 nm, (311) plane).

Ce-BTB@800 particles (Fig. S23) exhibited fragmentation after sonication. HR-TEM (Figures. 9b and S24) reveals randomly oriented CeO_2_ crystallites 2–6 nm in size, exposing (111), (100), and (222) planes with lattice spacings of 0.31, 0.28, and 0.32 nm, respectively. For Ce-TTB@800 (Fig. S25), HR-TEM (Figure 9c and Figure S26) shows surface CeO_2_ crystallites 4–12 nm in size, primarily indexed to the (222) and (311) reflections (0.32 nm and 0.33 nm).

### 3.4. OER activity of pyrolysed Ce(III)-MOFs

As with the pristine Ce(III)-based MOFs discussed above, the electrocatalytic OER activity of the pyrolysed Ce-MOF derivatives was investigated in 0.5 M aqueous H_2_SO_4_. The reaction kinetics and electrochemical stability of the cerium-based electrocatalysts under turnover conditions were evaluated using modified carbon paste (CP) electrodes in rotating disk electrode linear sweep voltammetry (RDE-LSV) experiments.

**Figure 10.**
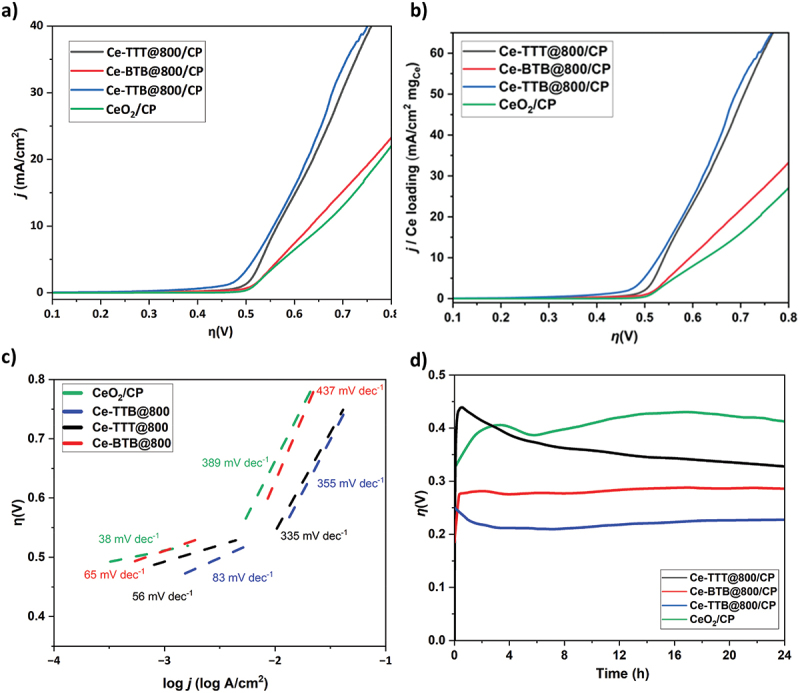
(a) RDE-LSV plot of pyrolysed Ce-MOFs, as well as CeO_2_ nanoparticles as benchmark electrocatalyst, embedded in carbon paste (CP) electrodes in 0.5 M H_2_SO_4_ electrolyte at a sweep rate of 1 mV s^−1^ and an electrode rotation speed of 1600 rpm. (b) RDE-LSV data normalised to Ce loading. (c) Tafel plot obtained from LSV data. (d) Chronopotentiometry data for pyrolysed Ce-MOFs and CeO_2_ nanoparticles embedded in carbon paste (CP) working electrodes. Measurements were performed at a constant current density of 1 mA cm^−2^ in 0.5 M H_2_SO_4_ electrolyte at an electrode rotation speed of 1600 rpm.

Figure 10a presents the RDE-LSV curves for each pyrolysed Ce-based catalyst, along with benchmark commercial CeO_2_. A comparison of the applied overpotentials (η) at various current densities is provided in Table 1. The onset overpotential, defined as the potential at which OER initiates, was estimated using the Tangent Method. Among the pyrolysed Ce-based catalysts, Ce-TTB@800/CP exhibited the lowest onset overpotential (338 mV), followed by Ce-TTT@800/CP (426 mV) and Ce-BTB@800/CP (450 mV). In comparison, the commercial electrocatalyst CeO_2_/CP displayed an onset overpotential of 472 mV.

The kinetics of the oxygen evolution reaction (OER) on the pyrolysed Ce-MOF electrodes were studied by Tafel plot analysis using data derived from rotating disk electrode linear sweep voltammetry (RDE-LSV) (Figure 10c). As was the case for the Ce-MOFs, the pyrolysed samples, presented in Figure 10, also exhibit two distinct Tafel regions, a lower overpotential region corresponding approximately to current densities of 1 mA cm^−2^ and a higher overpotential region corresponding approximately to current densities of 10 mA cm^−2^. As explained previously, only the lower overpotential region exhibits kinetic controlled behaviour and thus only Tafel slopes from this region are examine RDS. Among the pyrolysed materials, Ce-TTT@800/CP displays the lowest Tafel slope at 56 mV dec^−1^, followed by Ce-BTB@800/CP at 65 mV dec^−1^, both are consistent with a rate-determining chemical step [54–59]. Ce-TTB@800/CP shows a moderately higher slope of 83 mV dec^−1^ indicative of a chemical RDS under non-ideal (Temkin) coverage of intermediates or competition between a chemical and an electron transfer limiting step [54–59].

These values represent a moderate improvement over the kinetics observed for the pristine Ce-MOF precursors, which exhibited Tafel slopes of 81 mV dec^−1^ for Ce-TTT/CP, 87 mV dec^−1^ for Ce-BTB/CP, and 115 mV dec^−1^ for Ce-TTB/CP. Notably, the relative trend in kinetic performance is preserved following pyrolysis. This improvement, indicative of enhanced electron transfer, may be attributed to the presence of sp^2^-carbon domains within the pyrolysed Ce-MOFs, which enhance electronic conductivity, shifting the RDS towards the chemical step.

The long-term operational stability of the pyrolysed Ce-MOF electrocatalysts was evaluated using 24-hour chronopotentiometry experiments (Figure 10d). A constant current density of 1 mA cm^−2^ was selected based on the Tafel analysis (i.e. under kinetic controlled conditions) presented in Figure 10c.

All three pyrolysed Ce-MOF electrocatalysts appear to be relatively stable over the 24-hour chronopotentiometry experiment. For Ce-TTT@800/CP, a gradual decline in the applied overpotential is observed, decreasing from 440 mV at 30 minutes to 326 mV at 24 hours. This improvement in the overpotential required to maintain 1 mA cm^−2^ likely reflects the loss of the Ce_2_S_3_ phase (primarily within the first 5 hours) from electrode surface, exposing more cerium oxide active state. Ce_2_S_3_ rapidly hydrolyses in acidic aqueous environments and cannot partake directly in OER catalysis [80–82]. Ce-BTB@800/CP and Ce-TTB@800/CP also remained highly stable throughout the 24-hour chronopotentiometry experiment (Figure 10d), exhibiting overpotentials of 287 mV and 226 mV at 24 hours, respectively. The commercial CeO_2_ nanoparticles (CeO_2_/CP), included here as a benchmark, were also found to be relatively stable over the 24-hour test period (Figure 10d), with the overpotential increasing only slightly from 407 mV at 3 hours to 412 mV at 24 hours. Importantly, from 3 to 24 hours, all three pyrolysed Ce-MOFs outperformed the commercial CeO_2_ in terms of lower overpotentials. These findings suggest that the pyrolysis of Ce-based MOFs is a promising strategy for enhancing the electrocatalytic performance of CeO_2_-based nanomaterials.

To further access the long-term stability of the pyrolysed Ce-MOF/carbon paste electrodes for acidic OER, they were subjected to 1,000 CV cycles at a scan rate of 100 mV s^−1^ over a potential range of 0.2–2.1 V *vs*. SHE, in 0.5 M H_2_SO_4_. Figure S28a shows that Ce-TTT@800/CP exhibits robust OER activity throughout progressive cycling. After the 250th cycle Faradaic currents exhibit some decline with increasing cycle number, coinciding with an increasing capacitive current in the non-Faradaic region. This indicates the growth of an electrical double layer at the electrode-electrolyte interface. Given the complex nature of the electrode architecture (comprising a high surface MOF derivative embedded in carbon paste), this behaviour is not unexpected. At fast scan rates, diffusion within the electrode matrix does not reach steady state, resulting in capacitive charging during each cycle and mass-transported limited activity rather than catalyst degradation. This explanation is supported by LSVs recorded before and after 1,000 CV cycles (Fig. S28b). The slower scan rate (1 mV s^−1^) employed during LSV acquisition reduces capacitive contributions and shows that Ce-TTT@800/CP retains nearly identical OER activity after prolonged cycling. For instance, after 1,000 CV cycles, the electrode reaches a current density of 10 mA cm^−2^ at an overpotential of 565 mV, compared to 560 mV prior to cycling, a decay ratio of ~1%.

The stability of Ce-BTB@800/CP and Ce-TTB@800/CP observed under 1 mA cm^−2^ chronopotentiometric conditions contrast with the degradation seen during the harsher CV cycling stability experiments (Figure S29). For Ce-BTB@800/CP and Ce-TTB@800/CP, when capacitive current began to decline with increasing cycle number, so too did OER activity (Figures S29a and S29c, respectively). This reinforces the previously established correlation between mass transport-limited OER activity and increasing capacitive current during CV cycling. Under mass transport-limited OER conditions, the electrical double layer at the electrode-electrolyte interface becomes increasingly pronounced. When OER activity diminishes, this electrical double layer also collapses, as reactants and products diffuse into the bulk electrolyte and are not replenished at the electrode surface. As observed for Ce-BTB@800/CP and Ce-TTB@800/CP, the Ce(III)/(IV) redox couple (already located at the electrode surface) then accounts for the remaining Faradaic current recorded, which begins to decline after 500th cycle. This interpretation of CV data is confirmed my LSVs recorded at 1 mV s^−1^ before and after cycling (Figures S29b and S29c), where Ce-BTB@800/CP and Ce-TTB@800/CP show a marked decline in OER activity. Displaying increases in overpotential required to reach 10 mA cm^−2^ of 76 mV and 162 mV, respectively, corresponding to decay ratios of 12% and 29%.

It is well known that cerium oxide’s ability to catalyse various reactions is based on the accessibility of the Ce^3+^/Ce^4+^ redox couple and high oxygen mobility [37–43]. Increasing oxygen vacancies in CeO_2_ via the introduction of Ce^3+^, creating non-stoichiometric CeO_2-x_, is known to increase catalytic activity as the oxygen vacancies can act as the active sites [37–43]. The OER pathway is therefore likely via the lattice oxygen mechanism (LOM) [71–73]. Tafel slopes are consistent with LOM assignment. However, we acknowledge Tafel analysis alone cannot distinguish between LOM and AEM pathways. LOM assignment is supported by the cerium redox chemistry and likely defect structure of the operando phase rather than by kinetic analysis alone. Conclusive mechanistic assignment would require future in-situ ^18^O labelled differential electrochemical mass spectrometry (DEMS) experiments [71–73].

## 4. Conclusions

Ce(III)-based MOFs composed of one-dimensional chain SBUs were synthesised and characterised, then evaluated as electrocatalysts for the oxygen evolution reaction (OER) under acidic conditions. All Ce-MOFs functioned as pre-catalysts, undergoing *in situ* anodic oxidation to form catalytically active Cerium oxide phase. Among the pristine materials, Ce-TTT/CP exhibited the highest OER activity as evidenced by the LSV experiments, with performance comparable other non-noble metal electrocatalysts in the literature (see table S5). Chronopotentiometry @ 1 mA cm^−2^ revealed 24 h operational stability, while post-catalysis PXRD detected CeO_2_ as the dominant active phase.

Building on these observations, the MOFs were pyrolysed at 800°C to generate composite materials (Ce-TTT@800, Ce-BTB@800, and Ce-TTB@800) consisting of CeO_2_ nanocrystallites embedded in conductive carbon networks. Notably, pyrolysis of Ce-TTT produced both Ce_2_S_3_ and bulk defective CeO_2-x_ phases via *in situ* sulfurisation by CS_2_ derived from thiophene linker decomposition, representing the first MOF-based route to a lanthanide sulfide. Raman spectroscopy revealed high sp^2^-carbon contents (68–94%), consistent with enhanced conductivity and strong anchoring of cerium domains within the carbon matrix.

Pyrolysis substantially enhanced OER performance. At 10 mA cm^−2^, overpotentials were reduced by 56–179 mV relative to the parent MOFs, and improved OER kinetics with Tafel slopes decreased by up to 31% due the improved electron transfer courtesy of the sp^2^-carbon phases. All pyrolysed materials showed strong stability @ 1 mA cm^−2^ for 24 hours. Furthermore, Ce-TTT@800/CP exhibited excellent stability during 1,000 CV cycles with OER activity largely unaffected. While Ce-BTB@800/CP and Ce-TTB@800/CP partially deactivated under these harsh cycling conditions, the stability Ce-TTT@800/CP is likely due to the fact that the sulfurisation process introduced bulk defects to the ceria phase (consistent with anion-sublattice modification), in contrast to surface localised defects of Ce-BTB@800/CP and Ce-TTB@800/CP. This meant that even if some of the original surface of the Ce-TTT@800/CP ceria phase was lost during extended cycling, the newly exposed surface (formerly bulk) would contain the same amount of defects. This would not be the case for Ce-BTB@800/CP and Ce-TTB@800/CP.

Overall, this work demonstrates that Ce-MOFs are effective pre-catalysts and structural templates for generating active, acid-stable CeO_2_-carbon composites. The results highlight the key roles of linker-derived carbon structure, nanocrystallite anchoring, and controlled phase evolution in governing catalytic activity and durability. Future efforts should prioritise morphology optimisation to mitigate diffusion limitations and explore targeted doping strategies (e.g. Ir, Ru, Ti, Zr, or lanthanides) to further improve acidic OER performance while maintaining low precious-metal loadings. These design principles offer a viable pathway toward practical, earth-abundant alternatives to reduce Ir-loading on PEM electrolyser anodes.

## Supplementary Material

Supplemental Material

Supplemental Material
