# Supplementary material for: Ce(III)-MOFs with 1D chain architectures and their derivatives as earth-abundant electrocatalysts for the acidic oxygen evolution reaction
Source: Sci Technol Adv Mater. 2026 Jul 2;27(1):2694962. doi: 10.1080/14686996.2026.2694962 (PMC13439780; doi:10.1080/14686996.2026.2694962)

checkCIF/PLATON report

Structure factors have been supplied for datablock(s) Ce-TTT

THIS REPORT IS FOR GUIDANCE ONLY. IF USED AS PART OF A REVIEW PROCEDURE FOR PUBLICATION, IT SHOULD NOT REPLACE THE EXPERTISE OF AN EXPERIENCED CRYSTALLOGRAPHIC REFEREE.

No syntax errors found. CIF dictionary Interpreting this report

Datablock: Ce-TTT

Bond precision: C-C = 0.0200 A Wavelength=1.54178

Cell: a=21.7190(8) b=16.0619(8) c=8.1108(3) alpha=90
beta=94.903(4) gamma=90

Temperature: 100 K

	Calculated	Reported
Volume	2819.1(2)	2819.1(2)
Space group	C c	Cc
Hall group	C -2yc	C -2yc
Moiety formula	C21 H13 Ce N4 O7 S3, C3 H7 N O	C21 H13 Ce N4 O7 S3, C3 H7 N O, 1[]
Sum formula	C24 H20 Ce N5 O8 S3	C24 H20 Ce N5 O8 S3
Mr	742.75	742.77
Dx,g cm-3	1.750	1.750
Z	4	4
Mu (mm-1)	15.051	15.041
F000	1476.0	1480.6
F000’	1477.02	
h,k,lmax	23,17,8	23,17,8
Nref	3752[1880]	3652
Tmin,Tmax	0.835,0.860	0.530,0.751
Tmin’	0.471	

Correction method= # Reported T Limits: Tmin=0.530 Tmax=0.751

AbsCorr = NONE
Data completeness= 1.94/0.97 Theta(max)= 56.550

R(reflections)= 0.0731(2981) wR2(reflections)= 0.1906(3652)

S = 0.988 Npar= 367

The following ALERTS were generated. Each ALERT has the format **test-name_ALERT_alert-type_alert-level**. Click on the hyperlinks for more details of the test.

Alert level A

```
THETM01_ALERT_3_A  The value of sine(theta_max)/wavelength is less than 0.550
            Calculated sin(theta_max)/wavelength =    0.5412
```

Author Response: No diffraction seen past Two Theta 113.1 degrees. This is within expectations for a porous MOF.

```
PLAT027_ALERT_3_A _diffrn_reflns_theta_full value (too) Low ......      56.55 Degree
```

Author Response: No diffraction seen past Two Theta 113.1 degrees. This is within expectations for a porous MOF.

Alert level B

```
PLAT090_ALERT_3_B Poor Data / Parameter Ratio (Zmax > 18) ........       5.09 Note
```

Author Response: This is a false alert. According to Olex2 the real Data/Parameter ratio is 9.95, which is good.

Alert level C

```
RINTA01_ALERT_3_C  The value of Rint is greater than 0.12
            Rint given   0.142
PLAT020_ALERT_3_C The Value of Rint is Greater Than 0.12 .........      0.142 Report
PLAT042_ALERT_1_C Calc. and Reported MoietyFormula Strings  Differ     Please Check
              Calc: C21 H13 Ce N4 O7 S3, C3 H7 N O
              Rep.: C21 H13 Ce N4 O7 S3, C3 H7 N O, 1[]
PLAT068_ALERT_1_C Reported F000 Differs from Calcd (or Missing)...     Please Check
PLAT213_ALERT_2_C Atom C13             has ADP max/min Ratio .....        3.2 oblate
PLAT244_ALERT_4_C Low  MinorResAtom  Ueq as Compared to Neighbours        C23 Check
PLAT260_ALERT_2_C Large Average Ueq of Residue Including        O8      0.176 Check
PLAT342_ALERT_3_C Low Bond Precision on  C-C Bonds ...............       0.02 Ang.
PLAT369_ALERT_2_C Long   C(sp2)-C(sp2) Bond  C17      - C18       .      1.53 Ang.
PLAT601_ALERT_2_C Unit-Cell Contains Solvent Accessible VOIDS .LE.         32 Ang**3
```

```
PLAT911_ALERT_3_C Missing FCF Refl Between Thmin & STh/L=    0.541         12 Report
               -7  1  1,  -6  2  1,  -5  1  1,   1  1  1, -15 13  2, -11 15  2,
              -22  4  3, -14 12  4, -17  5  6,  10  0  6,  12  2  7,   5  5  8,
PLAT972_ALERT_2_C Check Calcd Resid. Dens.  0.70Ang From Ce1            -1.66 eA-3
PLAT975_ALERT_2_C Check Calcd Resid. Dens.  0.82Ang From O2      .       0.67 eA-3
PLAT976_ALERT_2_C Check Calcd Resid. Dens.  1.09Ang From O4      .      -0.68 eA-3
PLAT976_ALERT_2_C Check Calcd Resid. Dens.  1.04Ang From O7      .      -0.66 eA-3
PLAT976_ALERT_2_C Check Calcd Resid. Dens.  0.80Ang From O4      .      -0.64 eA-3
PLAT976_ALERT_2_C Check Calcd Resid. Dens.  0.94Ang From O6      .      -0.60 eA-3
```

Alert level G

```
PLAT002_ALERT_2_G Number of Distance or Angle Restraints on AtSite          5 Note
PLAT003_ALERT_2_G Number of Uiso or U(i,j)  Restrained non-H-Atoms         41 Report
PLAT004_ALERT_5_G Polymeric Structure Found with Maximum Dimension          3 Info
PLAT072_ALERT_2_G SHELXL First  Parameter in WGHT  Unusually Large       0.14 Report
PLAT073_ALERT_1_G H-atoms ref., but hydrogen treatment Reported as     constr Check
PLAT171_ALERT_4_G The CIF-Embedded .res File Contains EADP Records          1 Report
PLAT172_ALERT_4_G The CIF-Embedded .res File Contains DFIX Records          4 Report
PLAT173_ALERT_4_G The CIF-Embedded .res File Contains DANG Records          2 Report
PLAT174_ALERT_4_G The CIF-Embedded .res File Contains FLAT Records          1 Report
PLAT176_ALERT_4_G The CIF-Embedded .res File Contains SADI Records          1 Report
PLAT178_ALERT_4_G The CIF-Embedded .res File Contains SIMU Records          2 Report
PLAT187_ALERT_4_G The CIF-Embedded .res File Contains RIGU Records          2 Report
PLAT380_ALERT_4_G Incorrectly? Oriented X(sp2)-Methyl Moiety .....        C19 Check
PLAT380_ALERT_4_G Incorrectly? Oriented X(sp2)-Methyl Moiety .....        C24 Check
PLAT769_ALERT_4_G CIF Embedded Explicitly Supplied Scattering Data          6 Note
PLAT794_ALERT_5_G Tentative Bond Valency for Ce1       (III)     .       3.08 Info
PLAT802_ALERT_4_G CIF Input Record(s) with more than 80 Characters          3 Info
PLAT860_ALERT_3_G Number of Least-Squares Restraints ............         561 Note
PLAT909_ALERT_3_G Percentage of I>2sig(I) Data at Theta(Max) Still        53% Note
PLAT933_ALERT_2_G Number of HKL-OMIT Records in Embedded .res File         13 Note
               -7  1  1,  -6  2  1,  -5  1  1,  -5  3  1,  -4  2  1,  -3  1  1,
               -1  1 -1,   1  1 -1,   1  1  1,   3  1  1,   5  1 -1,   6  2 -1,
                7  1 -1,
PLAT948_ALERT_5_G Externally Supplied Scattering Factors       CIF          6 Note
PLAT969_ALERT_5_G The 'Henn et al.' R-Factor-gap value ...........      2.541 Note
              Predicted wR2: Based on SigI**2  7.50 or SHELX Weight 19.29
PLAT978_ALERT_2_G Number C-C Bonds with Positive Residual Density.          0 Info
PLAT982_ALERT_1_G The Ce-f'=   -1.8070 Deviates from  IT-Value =     -1.8482 Check
PLAT983_ALERT_1_G The Ce-f"=    9.6806 Deviates from  IT-Value =      9.6596 Check
PLAT983_ALERT_1_G The  S-f"=    0.5584 Deviates from  IT-Value =      0.5567 Check
PLAT994_ALERT_1_G SHELXL .ins Contains no or MERG 0 Instruction ..          ! Note
```

---

   2 **ALERT level A** = Most likely a serious problem - resolve or explain
   1 **ALERT level B** = A potentially serious problem, consider carefully
  17 **ALERT level C** = Check. Ensure it is not caused by an omission or oversight
  27 **ALERT level G** = General information/check it is not something unexpected

   7 ALERT type 1 CIF construction/syntax error, inconsistent or missing data
15 ALERT type 2 Indicator that the structure model may be wrong or deficient
   9 ALERT type 3 Indicator that the structure quality may be low
12 ALERT type 4 Improvement, methodology, query or suggestion
   4 ALERT type 5 Informative message, check

It is advisable to attempt to resolve as many as possible of the alerts in all categories. Often the minor alerts point to easily fixed oversights, errors and omissions in your CIF or refinement strategy, so attention to these fine details can be worthwhile. It is up to the individual to critically assess their own results and, if necessary, seek expert advice.

PLATON version of 15/01/2026; check.def file version of 02/01/2026

duplicate check

No duplication found

Datablock Ce-TTT - ellipsoid plot

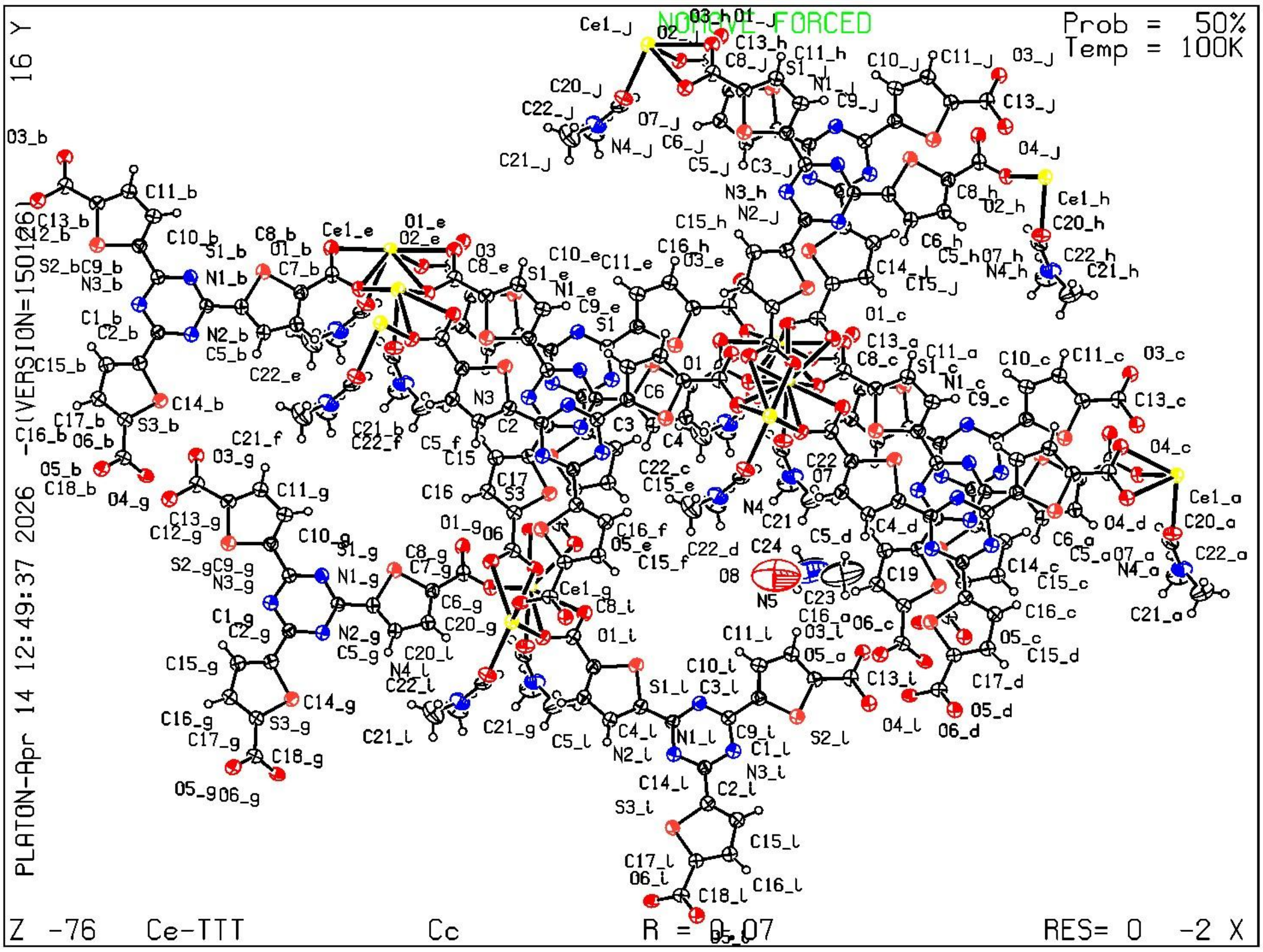

Supplement: Supplementary file 1 [file TSTA_A_2694962_SM7162.pdf]
